# Bioinformatics analysis of immune cell infiltration patterns and potential diagnostic markers in atherosclerosis

**DOI:** 10.1038/s41598-023-47257-8

**Published:** 2023-11-14

**Authors:** Haigang Ji, Ling Yuan, Wenbo Jiang, Yinke Jiang, Mengke Jiang, Xuemei Sun, Jing Chen

**Affiliations:** 1 Department of Cardiovascular Medicine, Changzhou Hospital Affiliated to Nanjing University of Chinese Medicine, Changzhou, 213003 China; 2 Department of Cardiovascular Medicine, Suqian Hospital Affiliated to Nanjing University of Chinese Medicine, Suqian, 223800 China; 3 Department of Gastroenterology, Tongde Hospital of Zhejiang Province, Hangzhou, 310012 China

**Keywords:** Computational biology and bioinformatics, Immunology, Biomarkers, Cardiology

## Abstract

This study aimed to investigate efficient diagnostic markers and molecular mechanisms of atherosclerosis and to analyze the role of immune infiltration through bioinformatics analysis. Expression profile datasets (GSE28829 and GSE43292) of patients with atherosclerosis and healthy controls were downloaded from the GEO database. Glutamine (GLN) metabolism-associated genes were obtained from the Molecular Signatures Database (MSigDB). The limma package in R was used to identify differentially expressed genes (DEGs). Significant modules were filtered using Weighted Gene Co-expression Network Analysis (WGCNA). MSigDB sets were subjected to Gene Set Enrichment Analysis and Gene Set Variation Analysis. The biological functions of DEGs were examined using Gene Ontology (GO) and Kyoto Encyclopedia of Genes and Genomes (KEGG) pathway enrichment analyses. STRING and Cytoscape software were used to identify hub genes and functional modules through protein–protein interaction (PPI) network analysis. The xCell software was adopted to assess the composition patterns of immune and stromal cells. Correlation analyses were performed for key genes and immune cell subtypes. We identified 308 DEGs and GLN-associated genes. Functional enrichment analysis showed that these genes were strongly enriched in muscle contract, muscle tissue development, cutile fiber, mycobacterial, and actin binding. Enriched KEGG pathways comprised dilated cardiomyopathy, hypertrophic cardiomyopathy, and the cAMP signaling pathway. In the PPI network analysis, 27 genes were identified as hub genes. The area under the curve (AUC) values of 27 biomarkers were relatively high, indicating high diagnostic values. The atherosclerosis group exhibited a markedly higher degree of infiltration than the control group. This study identified 27 GLN-associated genes as potential biomarkers for the diagnosis of atherosclerosis. It provides a new perspective on immune responses that facilitates exploration of the molecular mechanisms of atherosclerosis.

## Introduction

Atherosclerosis is a chronic inflammatory disease that affects the intima of the artery wall; it is a life-threatening manifestation of cardiovascular disease and presents as atherosclerotic rupture, chronic lumen stenosis, and thrombosis^1^. Cardiovascular diseases are the main cause of death globally and are primarily caused by atherosclerosis^2^. In general, treatment of atherosclerosis, initiated after the onset of symptoms of cardiovascular and cerebrovascular disease, aims to eliminate clinical symptoms. Therefore, early treatment of arteriosclerosis is an effective means of preventing cardiovascular and cerebrovascular diseases^3^. Hence, screening for marker genes is of critical importance for early diagnosis of atherosclerosis, for identifying new therapeutic targets, and for improving clinical therapeutic effects.

Glutaminolysis (GLN) suppresses oxidative stress and maintains the integrity of the mitochondrial membrane, facilitating cell survival^4^. GLN is a crucial energy source for immune and tumor cells. Nevertheless, inflammatory anti-tumor immune cells seem to exhibit no dependence on or even reject GLN metabolism, as evidenced particularly in macrophages^5^. M2 macrophages are more dependent on GLN than naïve macrophages, while M1 macrophages are characterized by inhibition of GLN metabolism. Hence, targeting GLN metabolism may serve as an essential strategy to shift tumor-associated macrophages from M2 to M1 phenotypes, thus augmenting anti-tumor immune responses^6^. Moreover, GLN metabolism is critically important in effector T cell activation and Th1 cell differentiation. Previous results indicate that targeting GLN metabolism has the potential to reshape the tumor microenvironment and enhance immunotherapy efficacy^7^. Indeed, extensive blocking of GLN metabolism greatly enhances the anti-tumor effects of anti-PD-1, while effector T cell cytotoxic function is also enhanced because of metabolic reprogramming^8^. Hence, it is crucial to target suitable metabolic pathways to block tumor metabolism and activate inflammatory immunity, thus improving immunization therapy. Targeting GLN metabolism represents a promising and potent option. GLN reduces the levels of atherosclerosis-related biomarkers and increases serum adiponectin levels, which may play an important role in reducing the occurrence and progression of atherosclerosis^9^. Therefore, GLN metabolism is an important component in disease occurrence.

Recently, bioinformatics methods have been broadly employed in high-throughput and microarray data assessments to identify differentially expressed genes (DEGs) and conduct a variety of research projects. Bioinformatics analysis has been recognized as a prominent method for identifying the underlying mechanisms of various human diseases. Based on an integrated genomic analysis of two public datasets, this study was conducted to explore the potentially important genes, key modules, infiltrating immune cells, and pathways involved in the pathogenesis of atherosclerosis.

## Results

A database in the Gene Set Enrichment Analysis** (**GSEA; http://www.gsea-msigdb.org/gsea/msigdb/index.jsp) platform^10,11^ was used to identify 134 GLN metabolism-associated genes.

### Weighted gene co-expression network analysis (WGCNA) and module screening

**Figure 1:**
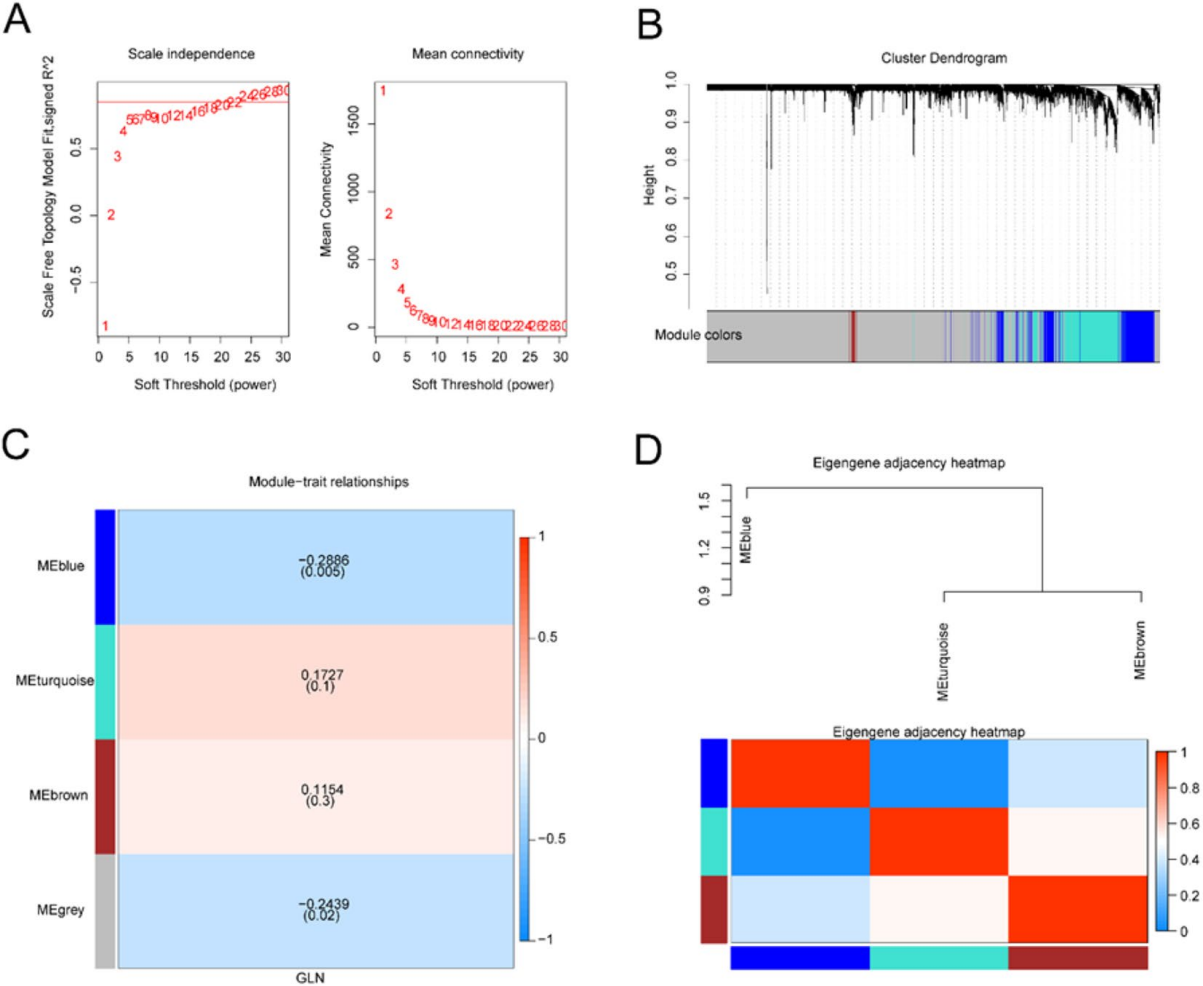
Establishment of Weighted Gene Co-expression Network Analysis** (**WGCNA). (**A**) Weight parameter β = 24 (soft threshold) and scale-free topology fitting index (R^2^). Various modules of co-expression data in atherosclerosis were identified using WGCNA. (**B**) Associations of modules. Top: Hierarchical clustering of module eigengenes (MEs) summarizing modules detected using clustering analysis. Branches of dendrogram groups (meta-modules) exhibited positive correlation with eigengenes. Bottom: Heatmap of adjacency relationships in the eigengene network. In the heatmap, there is correspondence between each row and column and one ME (colored), with red and blue indicating high and low adjacencies, respectively. Meta-modules refer to red squares along the diagonal. (**C**) Associations of consensus MEs with glutamine (GLN). In the table, there is correspondence between each row and consensus module, as well as between each column and a sample or trait. In the table, the numbers indicate the correlation coefficients for the corresponding ME and trait, and the corresponding *P*-values are printed in parentheses. The color legend indicates correlation coefficients. (**D**) Heatmap of overlapping gene network topologies. There is correspondence between each row and column and the gene in the heatmap, with light color and progressively darker red indicating low and higher overlapping topologies, respectively. Correspondence between modules and darker squares is observed along the diagonal. Dendrograms of genes and assignments of modules are exhibited on the left and top. (**F**) Correlation of gene significance (GS) with module membership (MM) for all GLN-associated genes in the blue module. ‘Cor’ refers to the absolute correlation coefficient of MM with GS.

GLN-associated gene sets were investigated using WGCNA. The results demonstrated that when the weighted value was 24 (Fig. 1A), scale independence was greater than 0.85 and mean connectivity was approximately 0. Three co-expressed modules were screened and the unrelated genes were distributed to a gray module, which was excluded from subsequent analyses (Fig. 1B). The module eigengenes (MEs) were correlated, thus allowing studying the associations among modules. A dendrogram and heatmap were adopted to depict the eigengene network (Fig. 1C). To understand the physiological significance of these modules, three MEs were associated with GLN, and the most significant associations were searched based on the heatmap of module-trait correlations (Fig. 1D).

### DEG identification

**Figure 2:**
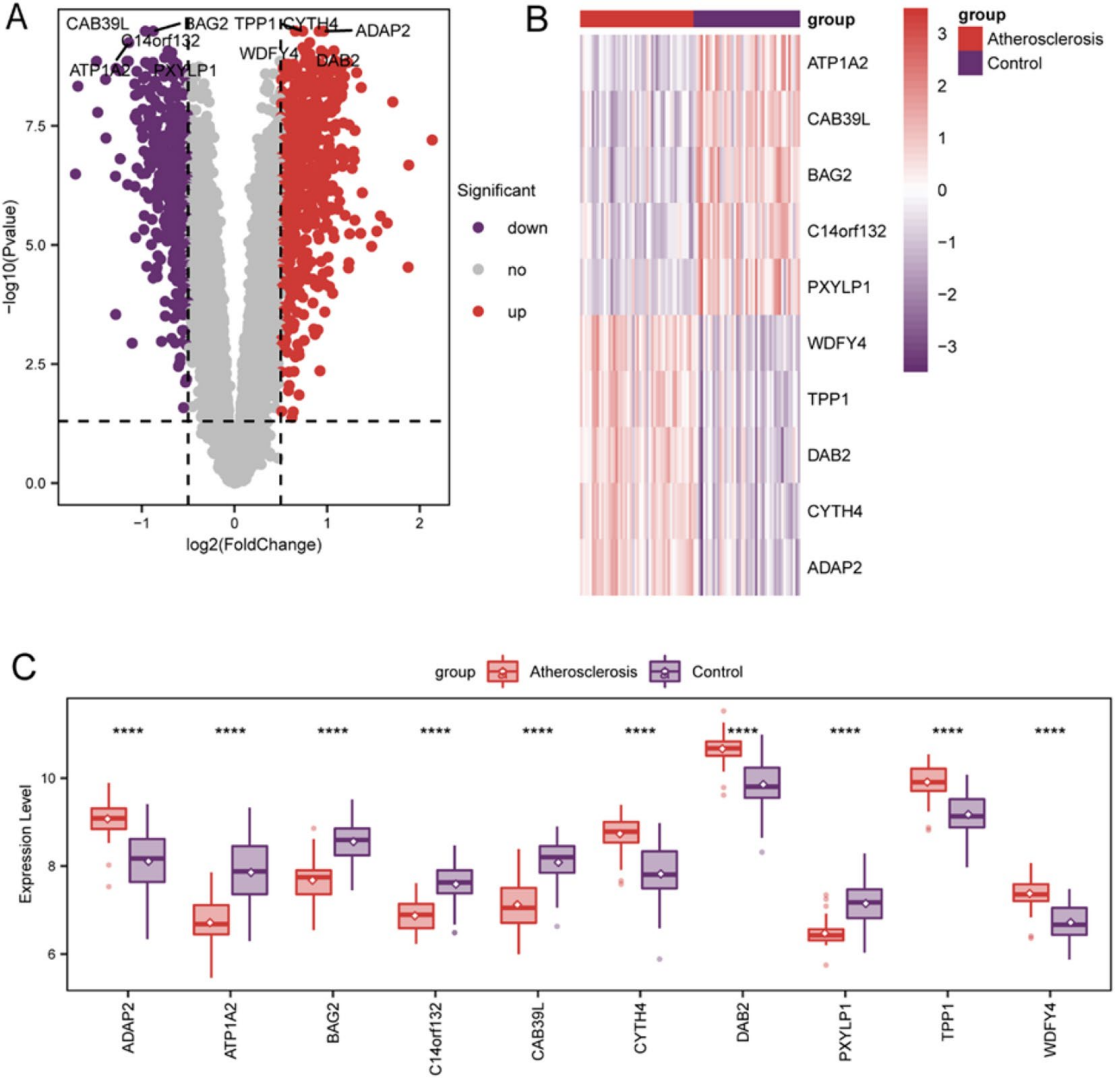
Identification of differentially expressed genes (DEGs). (**A**) Volcano plot of the distribution of DEGs in controls and atherosclerosis samples. Red dots: upregulated expression; purple dots: downregulated expression; gray dots: not significant expression. (**B**) Heatmap of the top 5 downregulated and upregulated DEGs. (**C**) The variations in the expression levels of 10 genes in both groups were revealed using Wilcoxon tests. Asterisks represent p values (*, **, ***, and **** represent p < 0.05, < 0.01, < 0.001, and < 0.0001, respectively).

A total of 1039 statistically significant DEGs were identified between healthy controls and atherosclerosis samples (p-adjusted < 0.05, |log2 fold change [FC]|> 0.5). Atherosclerosis samples included 619 genes with upregulated and 420 with downregulated expression. A volcano plot was generated for DEG visualization (Fig. 2A). The top 5 upregulated (*WDFY4*, *TPP1*, *DAB2*, *CYTH4*, and *ADAP2*) and downregulated DEGs (*ATP1A2*, *CAB39L*, *BAG2*, *C14orf132*, and *PXYLP1*) are shown using a heatmap (Fig. 2B). Based on the results of the Wilcoxon tests, these 10 genes showed marked differences in expression levels between atherosclerosis and control samples (p < 0.05, Fig. 2C).

We obtained 308 GLN-associated DEGs from the intersection between GLN-associated module genes and DEGs.

### GSEA

**Figure 3:**
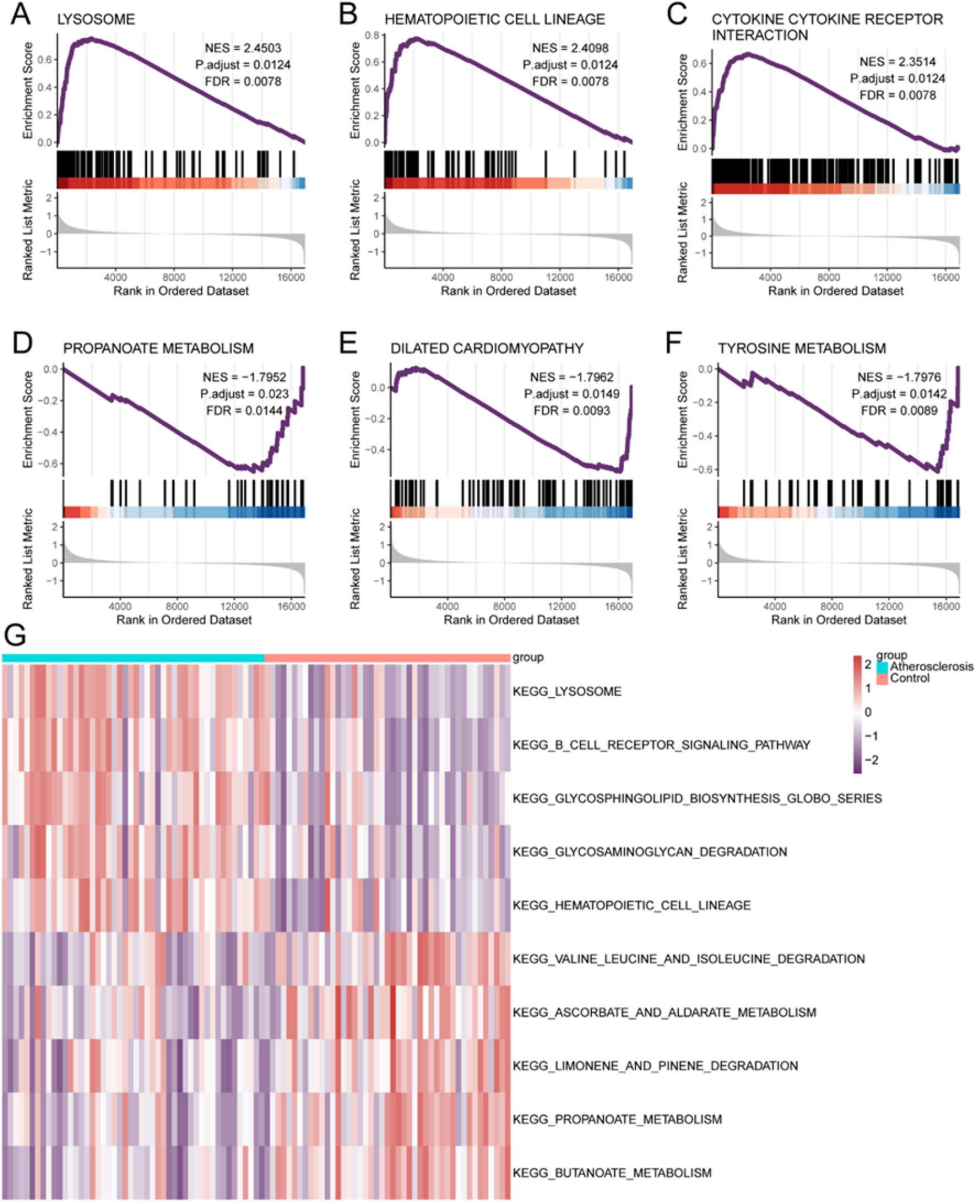
Gene Set Enrichment Analysis (GSEA) of significantly enriched pathways. (**A**) Lysosome, (**B**) hematopoietic cell lineage, (**C**) cytokine–cytokine receptor interaction, (**D**) propanoate metabolism, (**E**) dilated cardiomyopathy, and (**F**) tyrosine metabolism. (**G**) Heatmap illustrating the result of the GSVA analysis. *FDR* false discovery rate, *NES* normalized enrichment score.

To explore potential mechanisms underlying DEG function, GSEA was performed. A Molecular Signatures Database (MSigDB) collection was used to select the signaling pathways with the most significant enrichment according to the normalized enrichment score (NES). GSEA revealed that LYSOSOME (NES = 2.45, P-adjusted = 0.012, FDR = 0.008), HEMATOPOIETIC CELL LINEAGE (NES = 2.41, P-adjusted = 0.012, FDR = 0.008), CYTOKINE–CYTOKINE RECEPTOR INTERACTION (NES = 2.351, P-adjusted = 0.012, FDR = 0.008), PROPANOATE METABOLISM (NES =  − 1.795, P-adjusted = 0.023, FDR = 0.014), DILATED CARDIOMYOPATHY (NES =  − 1.796, P-adjusted = 0.015, FDR = 0.009), and TYROSINE METABOLISM (NES =  − 1.798, P-adjusted = 0.014, FDR = 0.009) (Fig. 3A–F) were significantly enriched in atherosclerosis.

### Gene set variation analysis (GSVA)

To further explore functional annotation, differences in the relative expression of pathways were evaluated between disease and control groups using GSVA. Numerous differentially expressed pathways were enriched and a heatmap was used for visualization. The disease group exhibited markedly lower expression of KEGG_LYSOSOME- and KEGG_HEMATOPOIETIC_CELL_LINEAGE-associated pathways, and markedly higher expression of KEGG_LIMONENE_AND_PINENE_DEGRADATION- and KEGG_BUTANOATE_METABOLISM-associated pathways than the control group (Fig. 3G).

### Enrichment analyses (Gene Ontology [GO]/Kyoto Encyclopedia of Genes and Genomes [KEGG])

**Figure 4:**
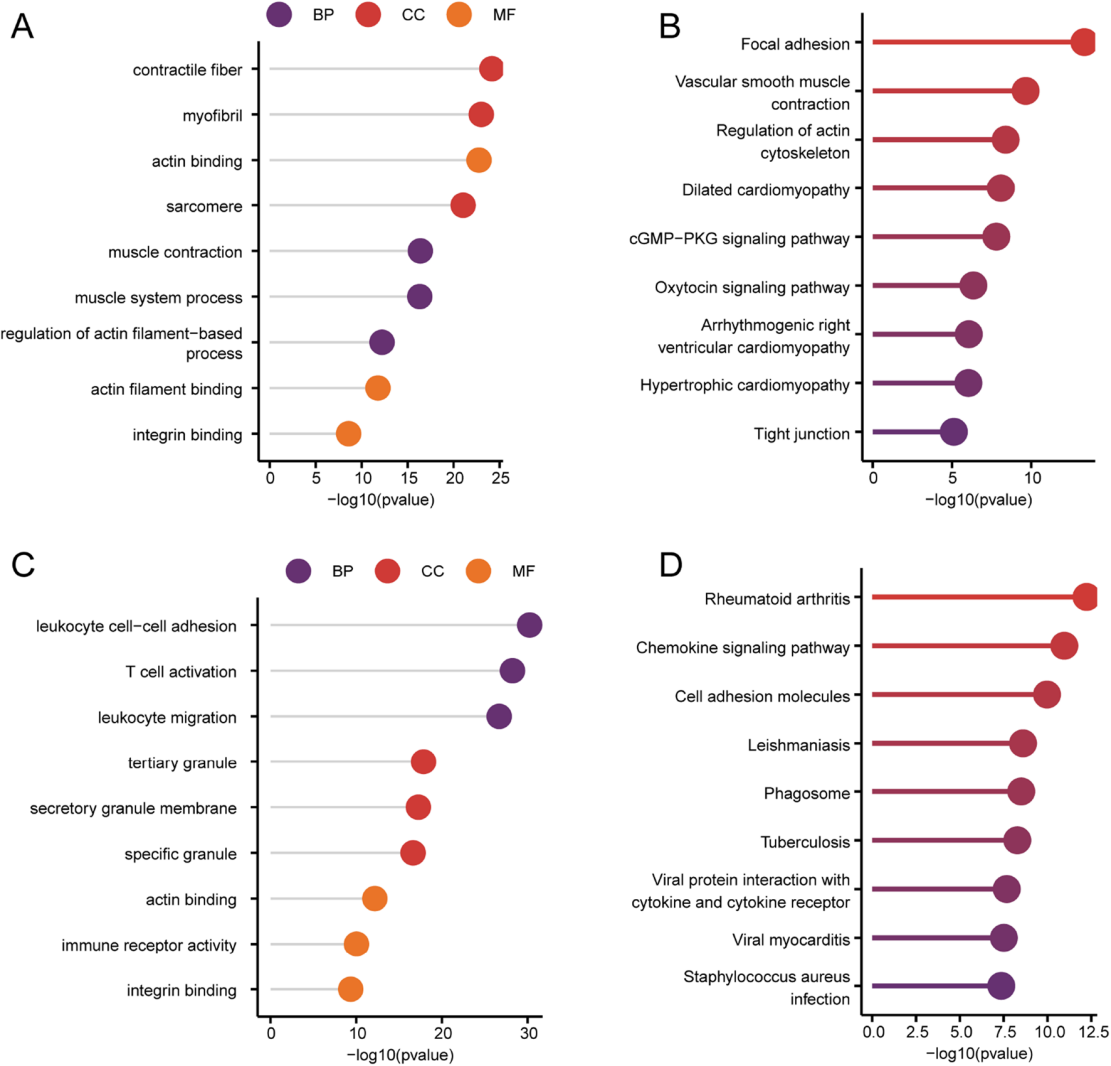
Enrichment analysis of the most significant module genes related to WGCNA and glutamine metabolism and the differential genes between atherosclerosis and normal controls. (**A**) The GO item enrichment analysis results of the most significant module genes related to WGCNA and glutamine metabolism are presented. (**B**) The KEGG enrichment analysis results of the most significant module genes related to WGCNA and glutamine metabolism are presented. (**C**) The enrichment analysis results of GO entries of differential genes between atherosclerosis and normal controls are displayed. (**D**) The KEGG enrichment analysis results of differential genes between atherosclerosis and normal controls were displayed.

To investigate the biological functions of the most significant module genes associated with WGCNA and glutamine metabolism compared with differential genes in atherosclerosis and normal controls, functional enrichment analysis was performed. GO results show, The most significant WGCNA and glutamine metabolism related module gene muscle contraction, muscle system process, regulation of actin filament—based process (BP), contractile fiber, myofibril, sarcomere(CC), actin binding, actin filament binding, integrin binding(MF) enrichment (Fig. 4A); The enriched KEGG pathways included Focal adhesion, Vascular smooth muscle contraction, Regulation of actin cytoskeleton, etc. (Fig. 4B). The GO results of differential genes in atherosclerosis and normal control showed that the genes were leukocyte cell–cell adhesion, T cell activation, leukocyte migration(BP), tertiary granule, secretory granule membrane, specific granule(CC), actin binding, immune receptor activity, integrin binding(MF) enrichment (Fig. 4C); Enriched KEGG pathways include Rheumatoid arthritis, Chemokine signaling pathway, Cell adhesion molecules and so on (Fig. 4D).

**Figure 5:**
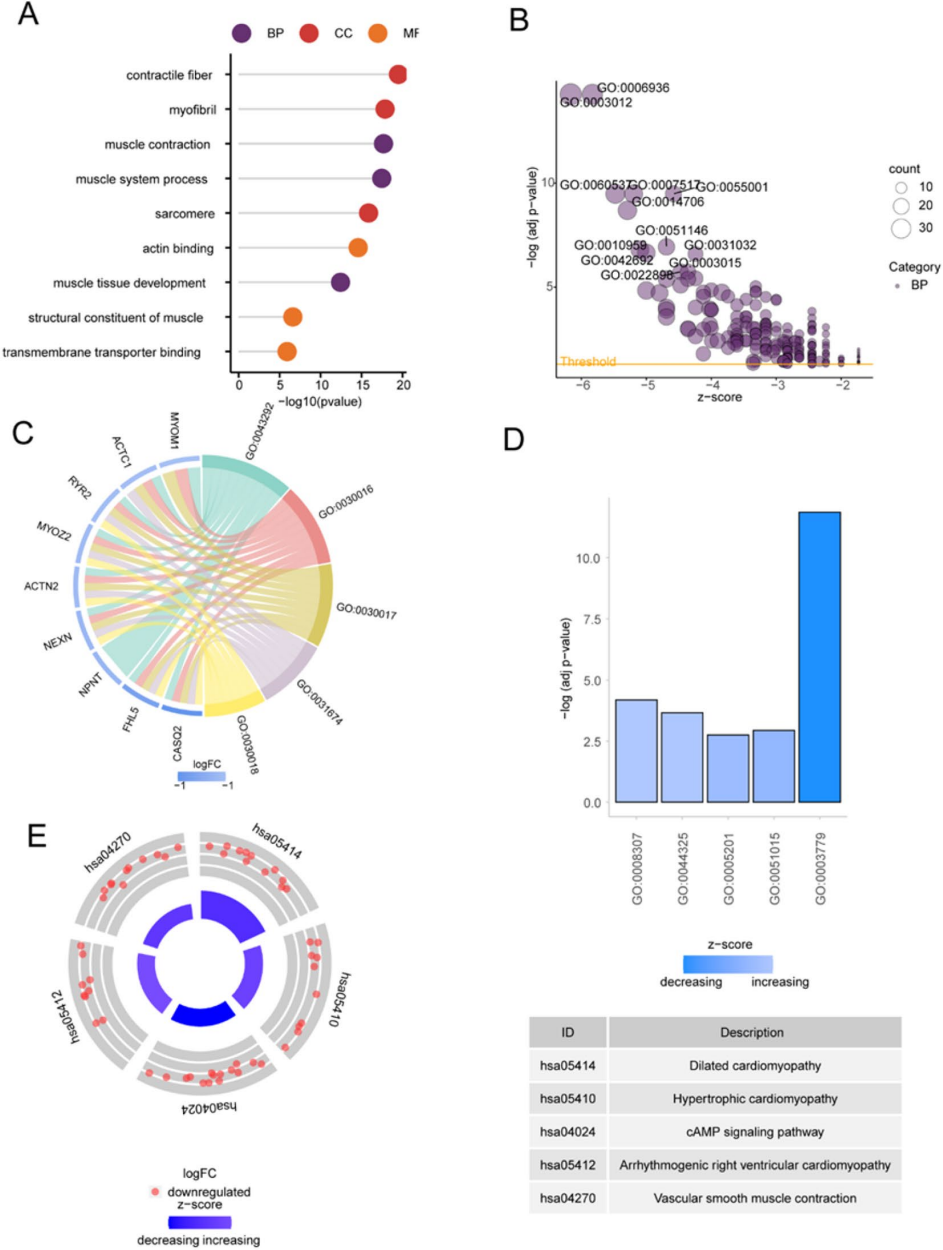
Functional enrichment analysis of GLN-associated DEGs. (**A**) Gene Ontology (GO) analysis marked terms. (**B**) Bubble chart of biological processes (BP). (**C**) Chord plot of cellular components (CC). (**D**) Bar plot of molecular functions (MF). (**E**) Circle plot showing pathway enrichment using Kyoto Encyclopedia of Genes and Genomes (KEGG) analysis of CC and pathway descriptions. *FC* fold change.

To investigate the biological functions of GLN-associated DEGs, GO term along with KEGG pathway enrichment analyses were performed. According to results of the GO term analysis, the genes exhibited strong enrichment in muscle contraction, muscle system process, and muscle tissue development (biological process [BP]); contractile fiber, myofibril, and sarcomere (cellular component [CC]); and actin binding, structural constituent of muscle, and transmembrane transporter binding (molecular function [MF]) (Fig. 5A–D).

The enriched KEGG pathways included dilated cardiomyopathy (DCM), hypertrophic cardiomyopathy (HCM), and cAMP signaling pathway (Fig. 5E).

### Protein–protein interaction (PPI) network analysis and hub gene screening

**Figure 6:**
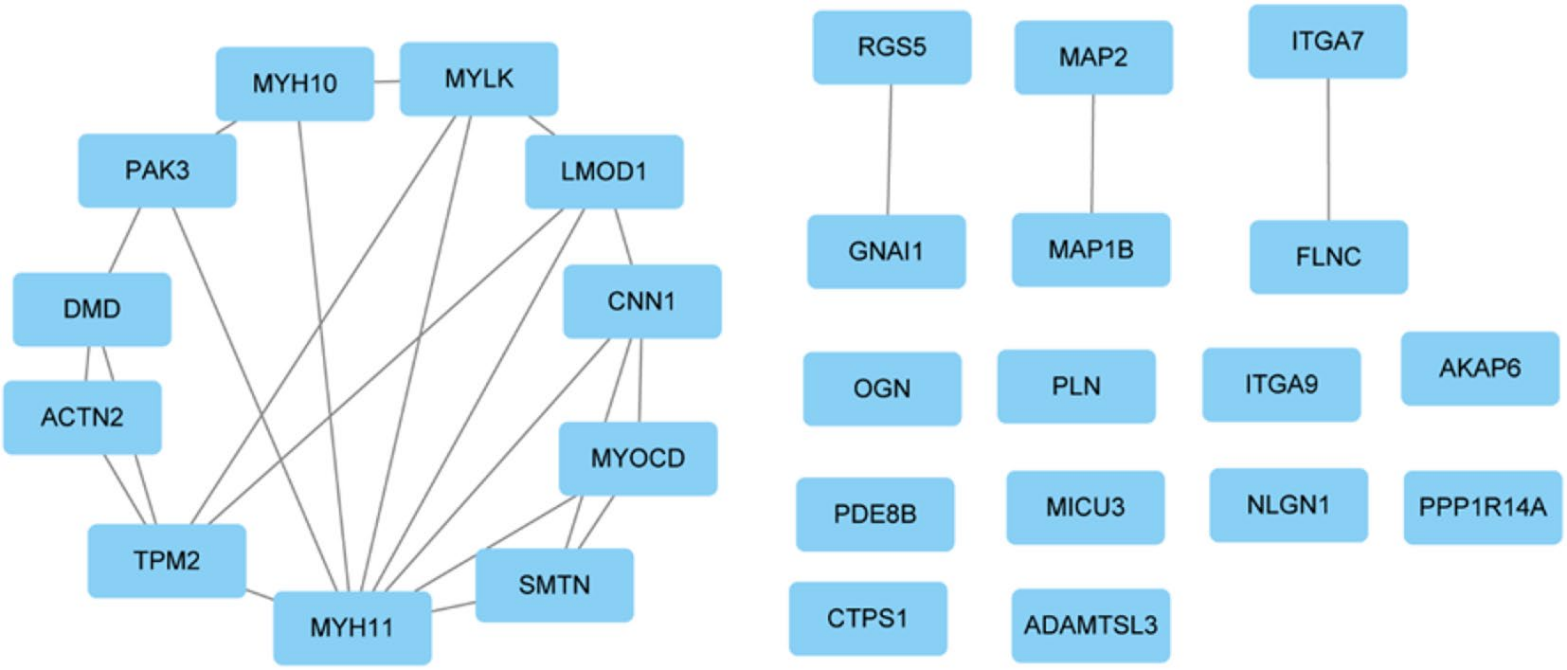
Hub genes of the protein–protein interaction (PPI) network. (**A**) Hub genes were screened based on gene intersections using 12 approaches.

To comprehend the interactions among the GLN-associated DEGs, a PPI network was built. Twelve approaches were adopted to elucidate highly correlated genes in the PPI network. Based on the intersections of the top 100 genes, obtained using all 12 methods, we identified 27 hub genes: *ACTN2*, *TPM2*, *FLNC*, *MYH11*, *ITGA7*, *DMD*, *PAK3*, *LMOD1*, *GNAI1*, *MYLK*, *PLN*, *CTPS1*, *CNN1*, *MYH10*, *NLGN1*, *ADAMTSL3*, *MYOCD*, *MICU3*, *PPP1R14A*, *MAP2*, *OGN*, *PDE8B*, *RGS5*, *MAP1B*, *ITGA9*, *AKAP6*, and *SMTN* (Fig. 6).

### Hub gene validation

**Figure 7:**
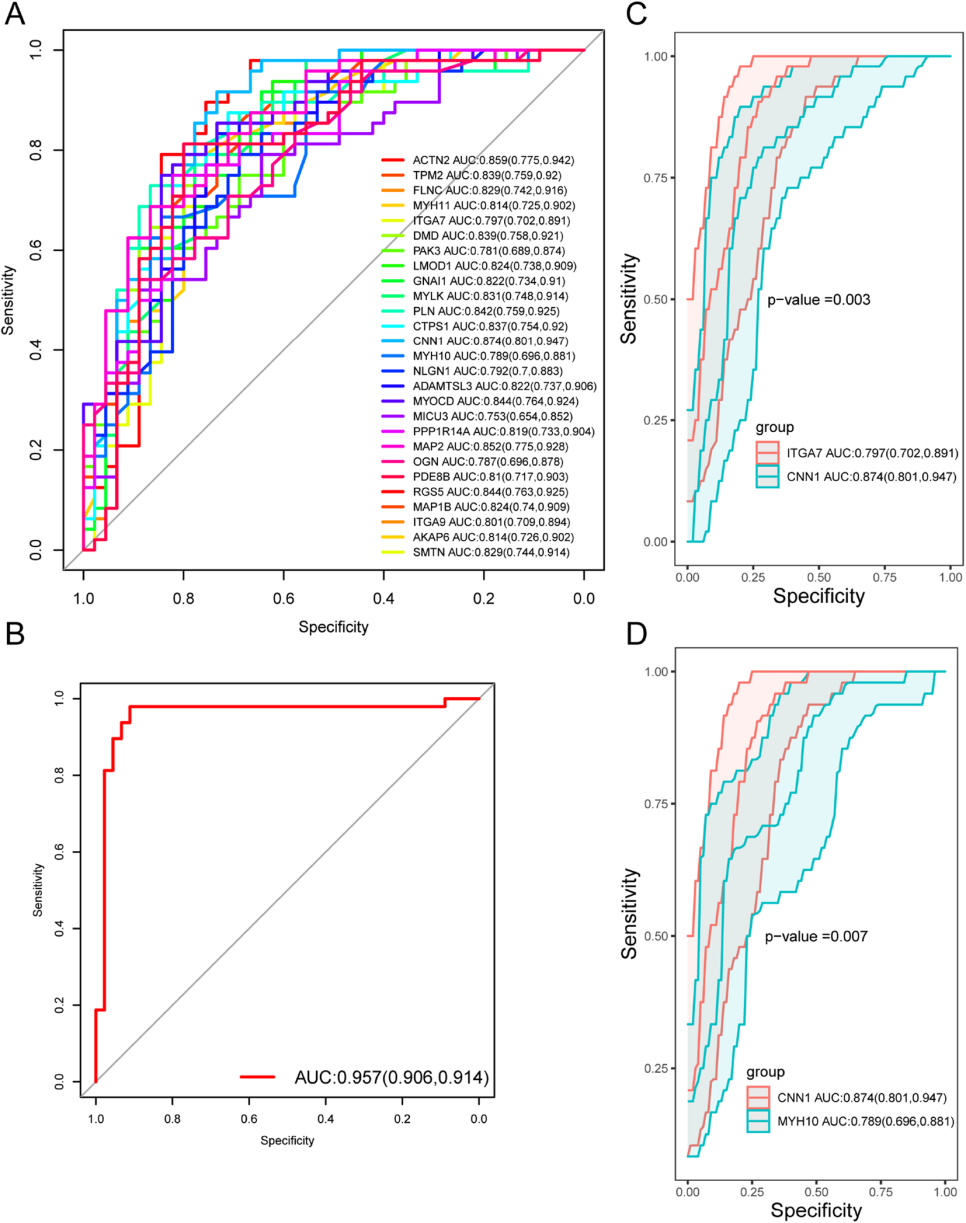
ROC curve analysis and testing of Hub gene. (**A**) ROC curve analysis of Hub gene. (**B**) 10 times cross validation ROC curve of AUC model. (**C**) ROC curves and delong test p-values of TGA7 and CNN1. (**D**) ROC curves and delong test p-values of MYH10 and CNN1.

To further verify the diagnostic value of hub gene, ROC curve was used to verify hub gene, and FLNC (AUC = 0.8287), AKAP6 (AUC = 0.8139), LMOD1 (AUC = 0.8236), DMD (AUC = 0.8394) were found. ACTN2 (AUC = 0.8588), GNAI1 (AUC = 0.8222), CTPS1 (AUC = 0.8368), MAP1B (AUC = 0.8241), ITGA7 (AUC = 0.7965), ITGA7 (AUC = 0.7965), the area under ROC curve (AUC) values of ADAMTSL3 (AUC = 0.8218) and ITGA9 (AUC = 0.8014) were both greater than 0.6 (Fig. 7A). We used tenfold cross-examination to verify the diagnostic efficacy of the AUC model, and found that the AUC value was 0.957 (Fig. 7B), indicating that the AUC model has good diagnostic efficacy, which indicates that the hub gene has the differential ability as a potential biomarker of atherosclerosis. Among them, CNN1 (AUC = 0.874 (0.801, 0.947)) has good diagnostic value. Next, we used delong test to detect whether there were statistical differences in ROC curves between CNN1 and other hub genes, and the results showed that most hub genes were significantly different from CNN1(p < 0.05), suggesting that CNN1 might be a biomarkers of atherosclerosis (Fig. 7C,D, Supplementary Fig. S1A–M).

### Immune cell infiltration (ICI)

**Figure 8:**
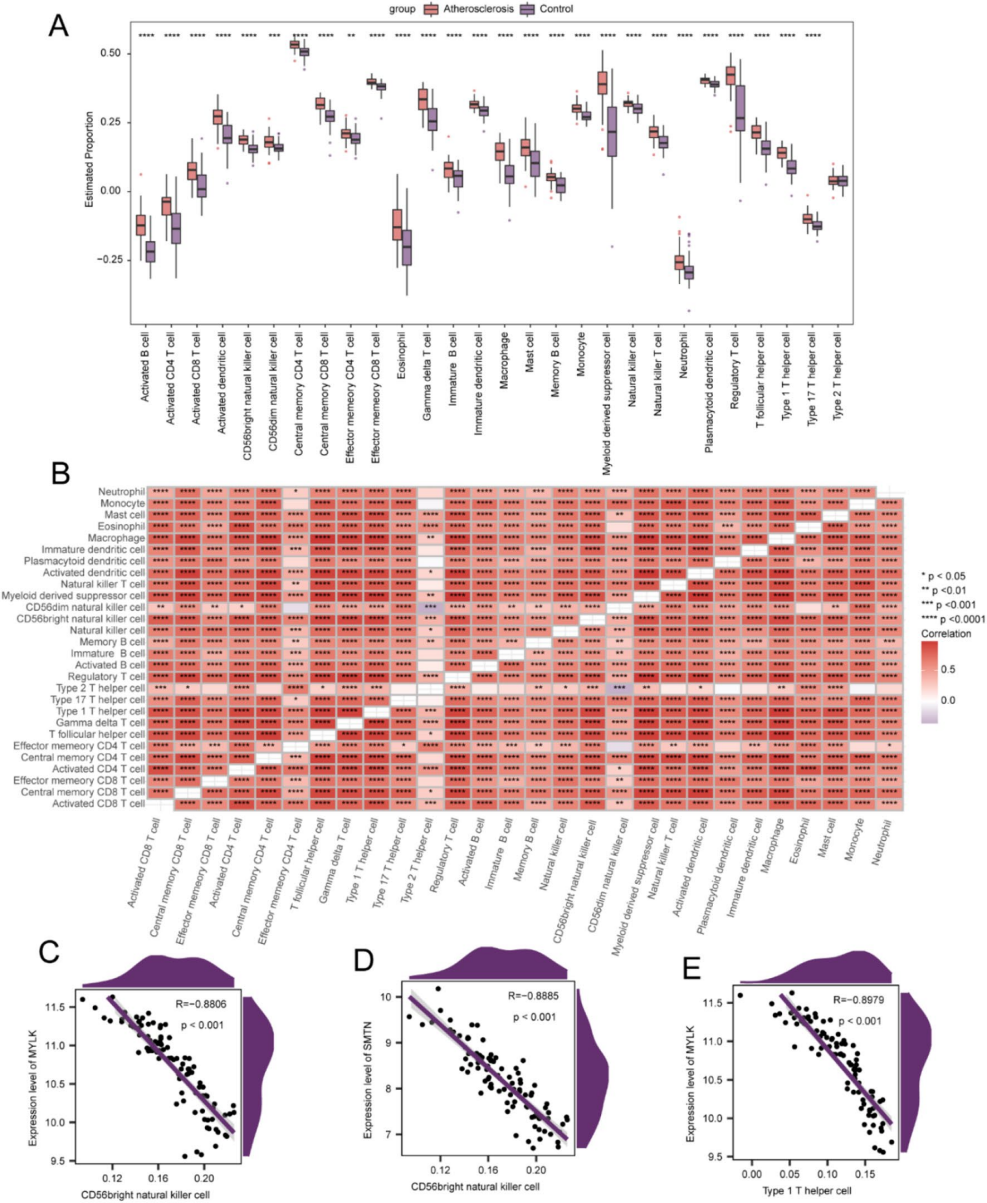
Immune cell infiltration (ICI) between atherosclerosis and control samples. (**A**) Heatmap of ICI changes in both groups. (**B**) Association among immune cells. Dot plots of the correlation between immune cells and genes *MYLK* (**C**), *SMTN* (**D**), and *MYLK* (**E**). *, **, ***, and **** represent p < 0.05, < 0.01, < 0.001, and < 0.0001, respectively.

Considering that ICI may play a crucial role in the pathogenesis of atherosclerosis, we examined the correlations between atherosclerosis/control samples and infiltrated immune cells. The atherosclerosis samples exhibited a markedly higher degree of infiltration than healthy controls, for most immune cells (Fig. 7A). A positive correlation was observed between the majority of immune cells (Fig. 8B). In addition, each hub gene showed a marked correlation with the corresponding immune cells (Fig. 8C–E). Notably, significant correlations were observed between *MYLK* and CD56bright natural killer (NK) cells (R = 0.881, *p* < 0.001), *SMTN* and CD56bright NK cells (R =  − 0.889, *p* < 0.001), and *MYLK* and Th1 (R =  − 0.898, *p* < 0.001) (Fig. 8C–E).

### Signaling pathways participated in signature genes

We used GSVA to examine the differences in 50 HALLMARK signaling pathways in atherosclerosis and control samples.

**Figure 9:**
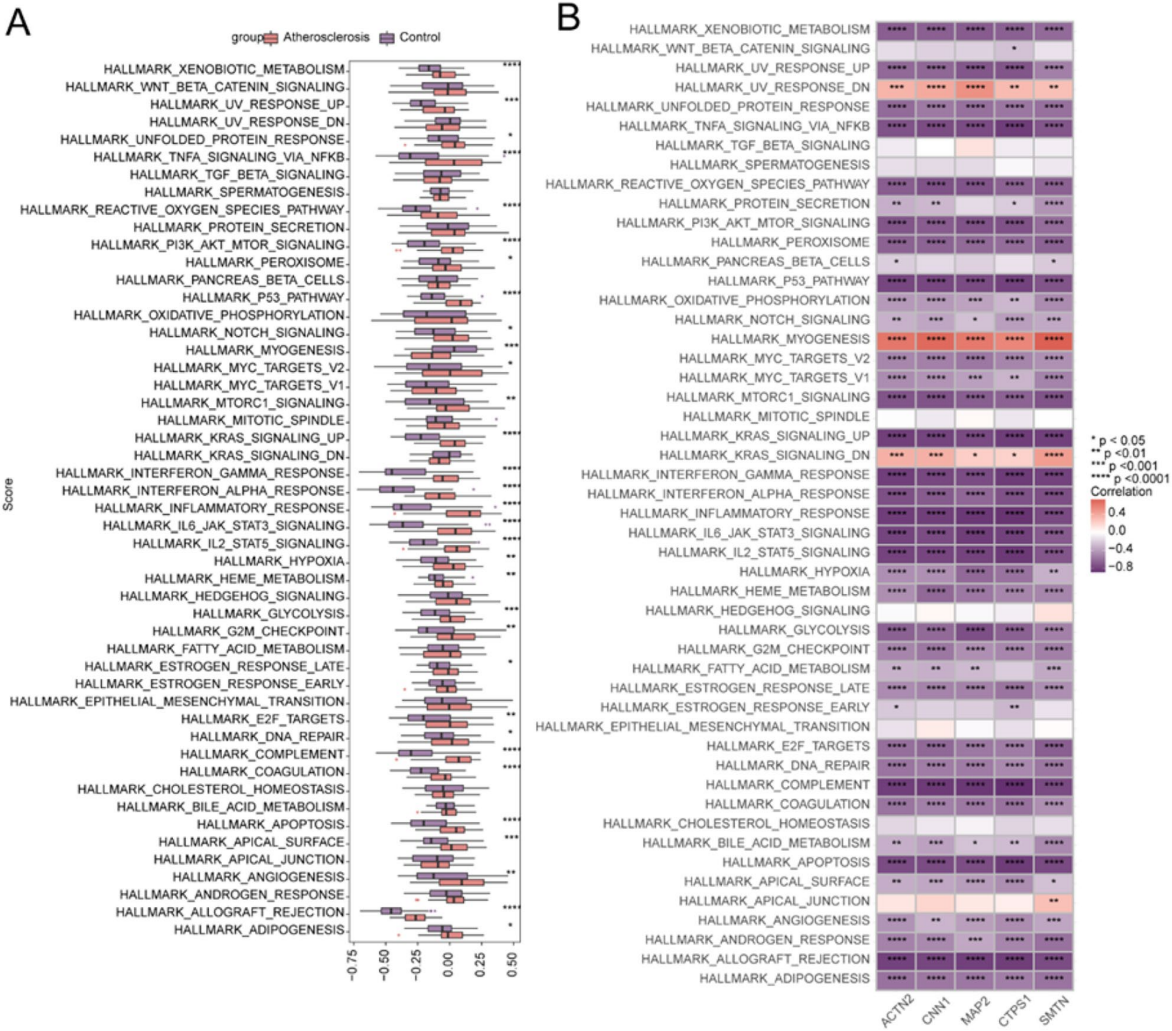
Associations of hub genes with 50 HALLMARK signaling pathways. (**A**) Fifty HALLMARK signaling pathways were compared in atherosclerosis and control samples. (**B**) Associations between 50 HALLMARK signaling pathways and target genes. *, **, ***, and **** indicate p < 0.05, < 0.01, < 0.001, and < 0.0001, respectively.

In the atherosclerosis samples, the expression of 31 HALLMARK signaling pathways was markedly upregulated: HALLMARK_ADIPOGENESIS, HALLMARK_ALLOGRAFT_REJECTION, HALLMARK_ANGIOGENESIS, HALLMARK_APICAL_SURFACE, HALLMARK_APOPTOSIS, HALLMARK_COAGULATION, HALLMARK_COMPLEMENT, HALLMARK_DNA_REPAIR, HALLMARK_E2F_TARGETS, HALLMARK_ESTROGEN_RESPONSE_LATE, HALLMARK_G2M_CHECKPOINT, HALLMARK_GLYCOLYSIS, HALLMARK_HEME_METABOLISM, HALLMARK_HYPOXIA, HALLMARK_IL2_STAT5_SIGNALING, HALLMARK_IL6_JAK_STAT3_SIGNALING, HALLMARK_INFLAMMATORY_RESPONSE, HALLMARK_INTERFERON_ALPHA_RESPONSE, HALLMARK_INTERFERON_GAMMA_RESPONSE, HALLMARK_KRAS_SIGNALING_UP, HALLMARK_MTORC1_SIGNALING, HALLMARK_MYC_TARGETS_V2, HALLMARK_NOTCH_SIGNALING, HALLMARK_P53_PATHWAY, HALLMARK_PEROXISOME, HALLMARK_PI3K_AKT_MTOR_SIGNALING, HALLMARK_REACTIVE_OXYGEN_SPECIES_PATHWAY, HALLMARK_TNFA_SIGNALING_VIA_NFKB, HALLMARK_UNFOLDED_PROTEIN_RESPONSE, HALLMARK_UV_RESPONSE_UP, HALLMARK_XENOBIOTIC_METABOLISM. Conversely, the expression of the HALLMARK_MYOGENESIS pathway was markedly downregulated in the atherosclerosis samples (Fig. 9A).

We also analyzed the correlations between the top 5 most-significant differentially expressed hub genes and 50 HALLMARK signaling pathways. *ACTN2* was associated with multiple pathways, including HALLMARK_UV_RESPONSE_UP and HALLMARK_UV_RESPONSE_UP (Fig. 9B).

### Interaction analysis on hub genes

**Figure 10:**
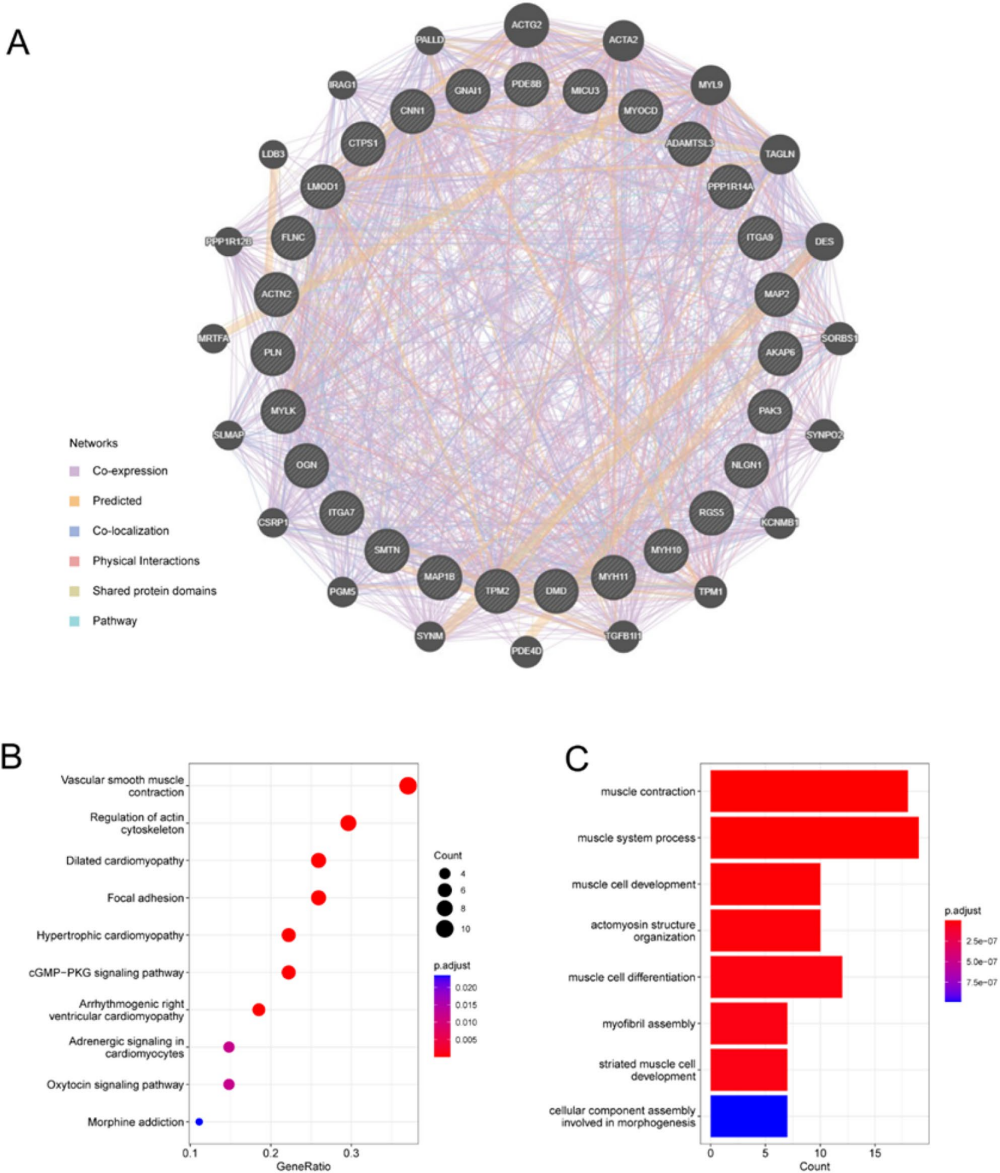
Interaction analyses of hub genes. (**A**) Co-expression network of signature genes. (**B**) GO and (**C**) KEGG analyses of co-expressed genes.

We created a PPI network for the signature genes using the GeneMANIA database and identified 27 genes (Fig. 10A). To further investigate the function of signature genes, GO and KEGG analyses were performed on 47 genes comprising 27 hub and 20 associated genes. According to the GO results, the genes were strongly enriched in regulation of axonogenesis, regulation of cell shape, and regulation of vascular smooth muscle (VSM) cell migration (BP); cell division site, cortical actin cytoskeleton, and caveola (CC); and cyclic-nucleotide phosphodiesterase activity, transcription coactivator activity, and transmembrane transporter binding (MF) (Fig. 10B). KEGG analysis revealed that DCM, VSM contraction, HCM, focal adhesion, regulation of actin cytoskeleton, arrhythmogenic right ventricular cardiomyopathy, adrenergic signaling in cardiomyocytes, cGMP-PKG signaling pathway, and oxytocin signaling pathway were the main enriched pathways (Fig. 10C).

### Validation of the gene set

**Figure 11:**
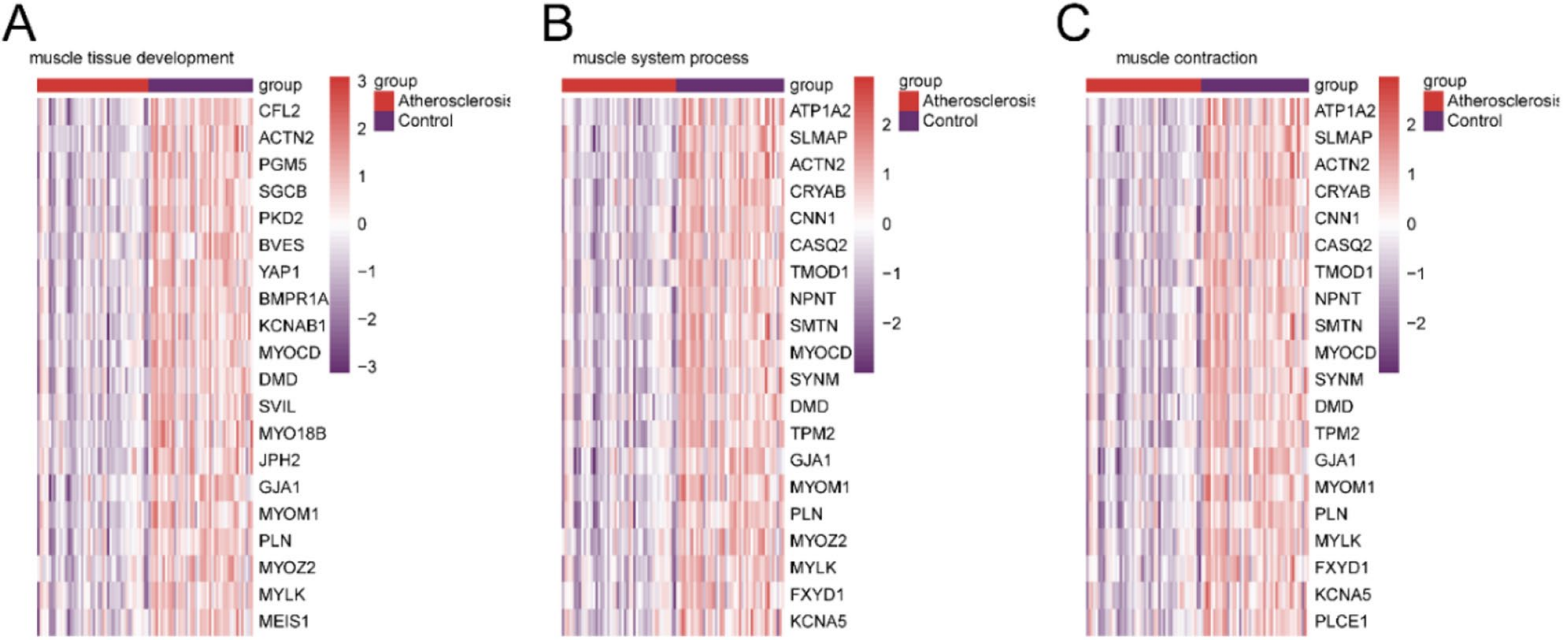
Heatmaps of key genes. Gene sets of muscle tissue development (**A**), muscle system process (**B**), and muscle contraction (**C**) in atherosclerosis and control samples.

The expression levels of the key genes validated enrichment for gene set muscle tissue development, muscle system process, and muscle contraction. The atherosclerosis samples displayed low expression levels for most of the key genes (Fig. 11).

## Discussion

As a chronic disease, atherosclerosis leads to ischemic symptoms in different sites^12^. Due to the absence of early diagnostic indicators, patients with atherosclerosis tend to miss optimal treatment opportunities, leading to adverse outcomes. Additionally, ICI has been reported to play a vital part in the development of atherosclerosis. Hence, it is crucial to identify specific diagnostic markers and analyze ICI patterns to improve the prognosis of patients with atherosclerosis. Using bioinformatics analysis, we identified diagnostic markers for atherosclerosis and investigated the role of ICI in this disease.

Among the 27 hub genes (*ACTN2*, *TPM2*, *FLNC*, *MYH11*, *ITGA7*, *DMD*, *PAK3*, *LMOD1*, *GNAI1*, *MYLK*, *PLN*, *CTPS1*, *CNN1*, *MYH10*, *NLGN1*, *ADAMTSL3*, *MYOCD*, *MICU3*, *PPP1R14A*, *MAP2*, *OGN*, *PDE8B*, *RGS5*, *MAP1B*, *ITGA9*, *AKAP6*, and *SMTN*) identified in this study, *ACTN2*, as pointed out earlier^13^, is a hub gene related to heart failure. *TPM2* has been identified as a potential diagnostic biomarker for atherosclerosis^14^. *FLNC* expression levels affect the TEAD-YAP/TAZ signaling pathway^15^. *ITGA7* serves as a tumor suppressor gene in breast cancer (BC) and modulates BC invasion and migration^16^. Moreover, the expression level of *ITGA7* in BC stem cells can be used as a predictor of chemotherapeutic efficacy in treating BC^17^. *PAK3* is a vital marker gene for the proneural subtype of glioma, affecting proliferation, differentiation, and growth^18^. As an oncogene related to Lauren classification, *LMOD1* modulates gastric cancer cell metastasis via the FAK-AKT/mTOR pathway^19^. These genes may serve as diagnostic or therapeutic targets for atherosclerosis, and their role should be confirmed through additional experiments.

ROC curve analysis revealed that the AUCs of several genes, including *FLNC* (AUC = 0.8287), *AKAP6* (AUC = 0.8139), *LMOD1* (AUC = 0.8236), *DMD* (AUC = 0.8394), *ACTN2* (AUC = 0.8588), *GNAI1* (AUC = 0.8222), *CTPS1* (AUC = 0.8368), *MAP1B* (AUC = 0.8241), *ITGA7* (AUC = 0.7965), *ITGA7* (AUC = 0.7965), *ADAMTSL3* (AUC = 0.8218), and *ITGA9* (AUC = 0.8014) were all high, indicating the outstanding diagnostic values of these genes. This implies that hub genes have a strong ability to distinguish between samples from patients with atherosclerosis and healthy controls. Further evaluation of these diagnostic genes based on an expanded sample size is required.

GO and KEGG annotation results demonstrated enrichment in pathways that regulate cell shape, axonogenesis, cell division site, caveola, transcription coactivator activity, and transmembrane transporter binding. VSM contractions and the cGMP-PKG signaling pathway are involved in the pathological mechanisms and potential treatments of heart failure^20^. STAT5 inhibitor can inhibit inflammation and alleviate atherosclerosis^21^. This was consistent with our GSVA results showing that atherosclerosis is closely related to IL2-STAT5-signaling. Then, Hsp27 might play an anti-atherosclerosis role by regulating apoptosis, which might provide a basis for the treatment of atherosclerosis^22^. Our GSVA results suggest that atherosclerosis is closely related to apoptosis, which is consistent with our results.

GSEA provides important information on large-scale genes with relatively small FC. By performing GSEA on the dataset gene profiles, we acquired multiple highly enriched gene sets in the atherosclerosis samples. Briefly, ‘LYSOSOME’ stands for genes modulated by lysosome-based signaling in response to acid phosphatase-type 5 (ACP5). The gene set comprised 33 genes, including Acp5 and cathepsin S (CTSS). Lysosomes participate in many biological processes, such as plasma membrane repair, immunity, cell adhesion and migration, gene regulation, and metabolic signal transduction^23^. Lysosome function and sphingolipid metabolism play important roles in the occurrence, development, and regulation of vascular disease and glomerular injuries^24,25^. Lysosome autophagy helps melanoma cells escape aging and promotes survival by recovering damaged organelles and proteins^26^. Lysosomes play important roles in many mechanisms related to atherosclerosis progression, including inflammation, exocytosis, autophagy, mTOR signal transduction, and iron metabolism^27^. Therefore, further studies on the roles of lysosome in the progression of atherosclerosis are required.

Genetic inactivation of PD-1–PD-L1 increases the burden of atherosclerosis and promotes the infiltration of macrophages and CD8 + T cells into atherosclerotic plaques^28^. To explore the function of ICI in atherosclerosis, an integrated assessment of immune infiltration was performed via single-sample GSEA (ssGSEA). The incidence of atherosclerotic cardiovascular events within 2 years after ICI treatment is 3 times higher^29^. Elevated infiltrated levels of activated B and CD4 T cells were observed. Correlation analysis with immune cells revealed that *MYLK* and *SMTN* were negatively correlated with CD56 + NK cells. NK cells play important roles in promoting tumor progression through immunosuppression^30,31^. Therefore, we speculate that *MYLK* and *SMTN* reduce CD56 + NK cell involvement in the occurrence and progression of atherosclerosis. This hypothesis requires further verification to elucidate the complex interactions of genes with immune cells.

## Conclusions

GLN is an important amino acid that provides carbon and nitrogen for biosynthesis. GLN is transported into cells through plasma membrane GLN transporters, such as SLC1A5, SLC38A1, and SLC38A217, and is then used in the cytoplasm for the biosynthesis of hexosamine, nucleotides, and asparagine^32^. GLN is also an essential nutrient for white blood cells (lymphocytes and macrophages), and GLN metabolic inhibitors are potential antiviral drug candidates^33^. Targeted GLN metabolism therapy can improve radiosensitization in prostate cancer^34^. Therefore, GLN metabolism plays a unique and important role in various diseases.

Briefly, DEGs, WGCNA and PPI modules, enriched pathways, hub genes, and infiltrated immune cells were examined, which may be particularly relevant to the pathogenesis of atherosclerosis. The results provide a novel perspective for understanding the pathogenesis of atherosclerosis and can drive progress in the development of therapeutics.

## Methods

### Data sources

The Gene Expression Omnibus (GEO; https://www.ncbi.nlm.nih.gov/geo/) database, which is freely accessible to the public, was used to derive all data used in our study. The atherosclerosis whole genome-wide expression profile was retrospectively derived from the GEO database using the R package ‘GEOquery’. GSE28829 comprises 16 samples from patients with atherosclerosis and 13 normal control samples. GSE43292 comprises 32 atherosclerosis samples and 32 control samples. The R package “sva” in ComBat method was applied to correct batch effects of non-biotechnical bias^35^. The degree of correction was examined using principal component analysis (PCA). The present study honored the data access policies of each database.

### DEGs associated with atherosclerosis

The “limma (version 3.50.0)”^36^ package in R was adopted to screen DEGs in atherosclerosis (n = 48) and normal (n = 45) samples, with thresholds of p-adjusted < 0.05 and |log2FC|> 0.5 for inclusion in subsequent evaluations. Next, the R package “pheatmap” was used to generate heatmaps by means of Complete Linkage Clustering and Euclidean Distance.

### WGCNA and significant module recognition

The WGCNA algorithm implemented in the WGCNA_1.70-3 package was adopted to build the co-expression networks^37^. Gene expression profile similarity was assessed using Pearson’s correlation coefficient. A scale-free network was obtained by weighing the correlation coefficients between genes via a power function. Using the R package ‘PickSoftThreshold,’ the similarity of co-expression was increased to power β = 24, thus building a weighted adjacency matrix. A gene module refers to a set of genes that are tightly interconnected in a co-expression network. In WGCNA, gene modules were identified using hierarchical clustering, and the modules are indicated in color. The different modules were screened using dynamic tree cut. During the module selection process, the adjacency matrix (a measure of topological similarity) was shifted to a topology overlay matrix (TOM), and cluster analysis was performed to detect modules. We implemented Pearson’s correlation analysis to calculate the correlation between ME (referring to the entire level of gene expression in each module) and GLN. Modules that were markedly related to GLN were acquired. Heatmap plots of topological overlap in the gene network were used to visualize the structure of the co-expression module. Hierarchical clustering dendrograms of MEs and heatmap plots of corresponding eigengene networks summarized the associations among modules. The GLN-associated DEGs were obtained from the intersections of DEGs and GLN-associated module genes.

### GSEA

As a computing approach, GSEA^11^ is used to examine if two biological states exhibit statistically significant and consistent differences in the a priori-defined set of genes. Using the R package “clusterProfiler (version 4.2.2),” GSEA was implemented on an ordered list of entire genes based on their log2FC values. Each analysis was conducted for 1,000 times of gene set permutations. We used c2.cp.kegg.v7.5.1.symbols as the reference gene collection from MSigDB^10,38^. Gene sets with P-adjusted < 0.05 were deemed as significantly enriched.

### GSVA

GSVA refers to an unsupervised and non-parametric approach that allows the utilization of gene expression profiles to evaluate connections between biological pathways and gene characteristics. To investigate the differences in biological function between normal and disease groups, GSVA was performed on “c2.cp.kegg.v7.5.1.symbols” using the R package “GSVA (version 1.42.0).” For result visualization, the R package “pheatmap (version 1.0.12)” was used. MSigDB (http://software.broadinstitute.org/gsea/msigdb) was employed to download 50 hallmark gene sets as reference. GSVA scores for the gene sets in various samples were computed using the ssGSEA function in the GSVA package. Both groups were compared in terms of GSVA score differences in various gene sets using the Limma package.

### GO/KEGG pathway enrichment analyses

GO^39^ divides gene functions into three parts: CC, BP, and MF. KEGG^40–42^ refers to an integrated bioinformatics platform for the systematic analysis of markedly altered metabolic pathways enriched in gene lists. The R package “clusterProfiler (version 4.2.2)”^43^ was employed for GO/KEGG enrichment analyses (p < 0.05) on the GLN-associated DEGs.

### PPI network establishment

We used the online database Search Tool for the Retrieval of Interacting Genes (STRING)^44^ to build PPI networks, setting 0.7 as the cut-off value of the interaction score. PPI network visualization was implemented using the Cytoscape^45^ software. In the PPI network, hub genes were detected via the Cytoscape plugin cytoHubba^46^. CytoHubba ranking methods were employed to screen the top 100 genes; hub genes were identified based on intersections from 12 approaches (Betweenness, BottleNeck, ClusteringCoefficient, density of maximum neighborhood component, edge percolated component, Eccentricity, maximum neighborhood component, maximal clique centrality, node connect closeness, node connect degree, Radiality, and Stress). The 12 methods in the CytoHubba plugin were used sequentially.

### GeneMANIA

The GeneMANIA website (http://genemania.org)^47^ was used to establish the PPI networks of hub genes. The website allows predictions of associations of functionally similar genes with hub genes, comprising protein–protein and protein–DNA interactions, pathways, biochemical reactions, and co-localization and co-expression networks. Based on the R package “clusterProfiler,” we obtained gene functional annotations of GO terms and KEGG pathway analyses.

### ROC curve

The ROC curve assesses diagnostic test performance. The curve is plotted with sensitivity as the ordinate and 1 − specificity (false positive rate) as the abscissa. AUC is a metric frequently acquired from the ROC plot of sensitivity against 1 − specificity. After using the R package “pROC” [PMID: 21414208] to plot ROC curves and determine AUC, the signature genes were screened and diagnostic values were assessed. AUC values ranged between 0.5 (completely random classifier) and 1 (perfect classifier). Generally, an AUC value of 0.5 refers to no predictive value, 0.6–0.8 to acceptable accuracy, 0.8–0.9 to excellent accuracy, and a value over 0.9 denotes outstanding accuracy.

### Immune infiltration analysis

SsGSEA^48^ refers to a GSEA extension. It computes the enrichment score for each sample paired with a gene set. The score indicates the absolute gene set enrichment within a specified dataset. SsGSEA differs from group-based (e.g., disease vs. normal) GSEA. Instead, each sample can be scored for the corresponding set of genes in ssGSEA.

We adopted the Tumor and Immune System Interactions Database (http://cis.hku.hk/TISIDB/index.php)^49^ to derive 28 immune cells: central memory CD4 and CD8 T cells, effector memory CD4 and CD8 T cells, activated CD4 and CD8 T cells, T follicular helper cells, gamma delta T cells, T helper cells (Th1, Th2, and Th17), regulatory T cells, activated B cells, immature B cells, memory B cells, NK cells, CD56bright and CD56dim NK cells, myeloid derived suppressor cells, NK T cells, activated dendritic cells, plasmacytoid dendritic cells, eosinophils, immature dendritic cells, mast cells, macrophages, monocytes, and neutrophils. The relative enrichment score of each immunocyte was quantitatively determined from the gene expression profile of each sample. Variations in the ICI levels among samples in atherosclerosis and control groups were illustrated using the R package ggplot2 (version 3.3.6)^50^.

### Statistical methods

The R software v4.1.2 was used to carry out the statistical analyses. The correlation between two parameters was inferred using Spearman’s correlation analysis. Inter-group differences were compared using Wilcoxon tests. Multi-group (three or more groups) differences were compared using the Kruskal–Wallis test. Statistical significance was set at two-sided p < 0.05.

### Supplementary Information

Supplementary Figure 1.

## Data Availability

The datasets used and/or analysed during the current study are available from the corresponding author on reasonable request.

## Abbreviations

AUC: Area under the curve
BC: Breast cancer
BP: Biological process
CC: Cellular component
CTSS: Cathepsin S
DCM: Dilated cardiomyopathy
DEGs: Differentially expressed genes
FC: Fold change
FDR: False discovery rate
GEO: Gene expression omnibus
GLN: Glutamine
GO: Gene ontology
GSEA: Gene set enrichment analysis
GSVA: Gene set variation analysis
HCM: Hypertrophic cardiomyopathy
ICI: Immune cell infiltration
KEGG: Kyoto encyclopedia of genes and genomes
ME: Module eigengene
MF: Molecular function
MSigDB: Molecular signatures database
NES: Normalized enrichment score
NK: Natural killer
PCA: Principal component analysis
PPI: Protein–protein interaction
ROC: Receiver operating characteristic
ssGSEA: Single-sample GSEA
STRING: Search tool for the retrieval of interacting genes
TCA: Tricarboxylic acid cycle
TOM: Topology overlay matrix
VSM: Vascular smooth muscle
WGCNA: Weighted gene co-expression network analysis
