# Supplementary figures and images for: Bioinformatics analysis of immune cell infiltration patterns and potential diagnostic markers in atherosclerosis
Source: Sci Rep. 2023 Nov 14;13:19821. doi: 10.1038/s41598-023-47257-8 (PMC10645850; doi:10.1038/s41598-023-47257-8)

Supplementary Figure 1.


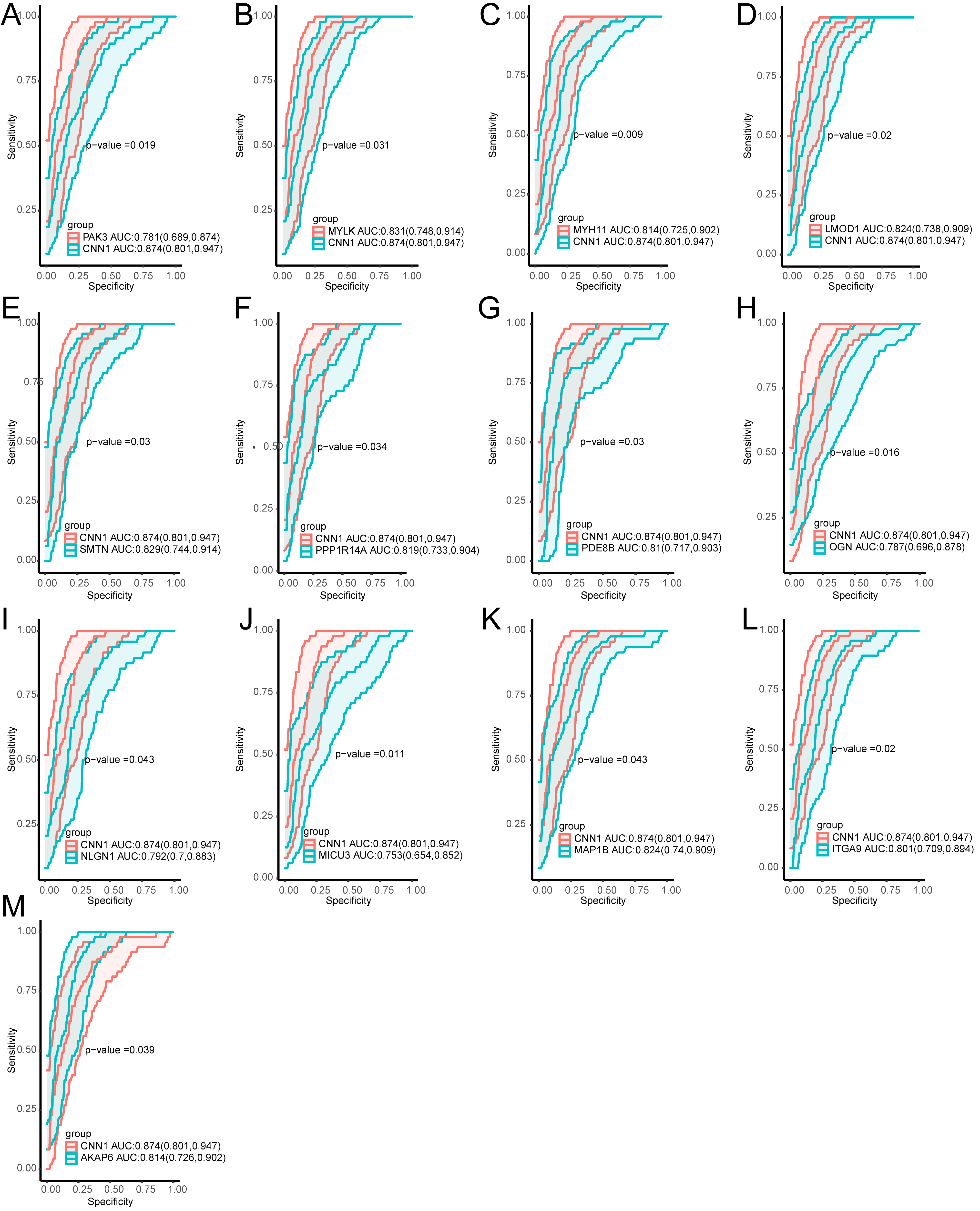

Supplement: Supplementary file 1 — Supplementary Figure 1. [file 41598_2023_47257_MOESM1_ESM.docx]
